# Mothers’ Experiences with Child Protection Services: Using Qualitative Feminist Poststructuralism

**DOI:** 10.3390/nursrep11040084

**Published:** 2021-11-15

**Authors:** Megan Aston, Sheri Price, Martha Paynter, Meaghan Sim, Joelle Monaghan, Keisha Jefferies, Rachel Ollivier

**Affiliations:** 1 School of Nursing, Dalhousie University, Halifax, NS B3H 4R2, Canada; sheri.price@dal.ca (S.P.); mpaynter@dal.ca (M.P.); cmonaghan@dal.ca (J.M.); keisha.jefferies@dal.ca (K.J.); rachel.ollivier@dal.ca (R.O.); 2 Nova Scotia Health, Research, Innovation and Discovery, Halifax, NS B3H 2E2, Canada; meaghan.sim@nshealth.ca

**Keywords:** postpartum, child protection, community, family resource centers, feminist, poststructuralism, qualitative, discourse analysis

## Abstract

Background: The postpartum period is often portrayed as a blissful, calm and loving time when mothers, partners and family members bond with their newborn babies. However, this time may be experienced quite differently when mothers are monitored by Child Protection Services. Having a baby under these circumstances can be very difficult and traumatizing. While all new parents require support and information to help them through the transition to parenthood and address physical and psycho-social changes, mothers who are involved with Child Protection Services require more specialized support as they encounter higher incidences of postpartum stressors and higher rates of poverty, mental illness and substance abuse. The impact of support for mothers involved with Child Protection Services is not well-understood from the perspective of mothers. Aim: The aim of the study was to understand how new mothers in Nova Scotia prioritized their postpartum needs and where they went to obtain information and support. Methods: Feminist poststructuralism was the methodology used to understand how the experiences of five mothers who accessed a family resource center and had been involved with Child Protection Services in Nova Scotia Canada had been personally, socially and institutionally constructed. Results: Themes include: (1) We are Mothers, (2) Being Red Flagged, (3) Lack of Trust, (4) Us Against Them and (5) Searching for Supportive Relationships. Conclusion: Personal stories from all participants demonstrated how they experienced stigma and stereotypes from healthcare workers and were often not recognized as mothers. They also struggled to find information, supports and services to help them keep or regain their babies.

## 1. Introduction

Having a new baby is a transformative time for mothers, partners and other family members. Images of new parents and babies usually depict loving embraces of caring, blissfulness and calm. However, the early postpartum period may look quite differently for those who are dealing with complex psycho-social and physical changes [1,2,3] that require specific support, information and intervention. One example of these complex psycho-social changes may include postpartum depression, which is the most common complication of pregnancy [4]. The postpartum period can be especially difficult and even traumatizing for mothers when being monitored by Child Protection Services and are fearful that they may have their baby taken away [5,6]. The incidence of postpartum stressors and instability are noted to be much higher for mothers who are involved with Child Protection Services—many of whom experience systemic and chronic disadvantages including poverty, mental illness and substance use [6]. Stemming from racism and colonialism, parents who are Indigenous or Black are also disproportionately represented in the Child Protection Services system [7,8].

To cope with stressors in the postpartum period, mothers often search for information and support from a variety of sources, including public health departments, formal support groups, community drop-in centers, online chat spaces, social media, family and friends [9,10,11,12,13,14]. However, it is difficult to determine the degree to which these programs and supports effectively address the wide spectrum of urgent and ongoing needs of new mothers and their families, especially mothers who have been mandated by the justice system to enroll in parenting classes and work with Child Protection Services.

Researchers have found that new mothers have significant social support needs in the postpartum period, such as enabling mothers to build the confidence needed to effectively care for their newborn, as well as wanting to know when and where to seek help [15,16]. These researchers found that some mothers did not access formalized postpartum services beyond a few visits to their primary care provider, despite persistent needs such as difficulties with breastfeeding or a lack of social support [15]. In addition, new mothers who are involved with Child Protection Services are also distrusting of accessing formal postpartum services for fear of having their child apprehended [17]. Moreover, dealing with Child Protection Services has been identified by new mothers as a stigmatizing, depersonalized and traumatic process where mothers often feel judged and blamed by the healthcare and social service institutions [18,19]. Outside of formalized postpartum services, mothers have historically sought social connections, in person and online, to help cope with the challenges of becoming a mother [20]. However, there has been little research about how these resources, particularly online, are accessed and the degree to which mothers’ needs for information and support are met [21]. Marginalized new mothers have unique needs and considerations when accessing online and offline information and support and more research is needed to inform future initiatives that are accessible, reliable and trustworthy [22].

The aim of the study was to understand how new mothers in Nova Scotia prioritized their postpartum needs and where they went to obtain information and support. Our overarching research question was: How do first-time mothers identify and prioritize their own postpartum needs and where do they go to access information and support within the first six months postpartum? Our secondary question was to explore: How are first-time mothers’ experiences socially and institutionally constructed? For the purpose of this paper, we report findings from one focus group conducted at an urban family resource center, where the majority of participants had had their newborn babies removed from their care by Child Protection Services or were being monitored by Child Protection Services. These findings are a subset of a larger qualitative study that aimed to examine how first-time mothers in Nova Scotia identified and prioritized their own postpartum needs and where they sought information and support (both online and offline) [9].

## 2. Background

The World Health Organization has declared that maternal, child and newborn healthcare are global health issues [23]. In Canada, the government encourages provinces and territories to provide programs and services for all mothers and families, with emphasis on those facing difficult life circumstances [24]; however, many would argue that accessibility to postpartum services is lacking. At the provincial level, Nova Scotia guidelines “Healthy Babies, Healthy Families” were created to support the early postpartum transition of mothers and newborns [25]. The provincial Department of Health and Wellness programs include Public Health nurse telephone support and home visiting as well as an Enhanced Home Visiting program [26]. Postpartum services that are also available to mothers and their families include community-based services, family resource centers, supports provided by individual healthcare practitioners and grassroots programs.

Despite a variety of available government and community programs and services to support new mothers and families, not all mothers choose to use these services. Reasons for this lack of uptake remain unclear as this is a currently under-researched area. The federal, provincial and territorial governments have intensified their focus on the provision of care for ‘at-risk’ families, such as single parents, low socioeconomic status, racial/ethnic minorities and those who experience mental health or substance use issues [24,27]. This shift is based on research conducted globally, confirming that mothers identified as ‘at risk’ who receive services from public health and other healthcare providers experience positive health outcomes, such as reduced hospital admissions, successful breastfeeding and increased self-esteem [28,29,30,31,32,33,34,35,36,37,38,39].

Studies on the health outcomes of new mothers primarily focus on measures such as breastfeeding rates, hospital admissions/re-admissions and indicators of physical health attributed to both mother and baby [24]. Despite the extant research on maternal postpartum needs, there remains a lack of comprehensive understanding surrounding the information and support-seeking practices of first-time mothers, and in particular those who are most marginalized. Many mothers experience systemic barriers to adequate support and information, including racism [40], classism [41] and homophobia/transphobia [42]. Some mothers feel judged and unwelcomed in community groups [15] or lack financial means for transportation to services and programs [43]. In addition, given that public health services such as home visits have shifted resources to focus on mothers deemed ‘at risk’, universal services for all mothers have been significantly reduced [15,44]. Although it is important to ensure that services are delivered to those who need it most, our own research demonstrated how public health home visiting programs targeted for those ‘at risk’ are affiliated with pervasive stereotypes, stigmas and oppressive surveillance [15]. While all mothers may share similar needs (e.g., breastfeeding or mental health supports), mothers from ‘targeted’ groups expect to be judged by care providers due to their ‘at risk’ status [15]. Previous research found that public health nurses (PHNs) were aware of these tensions and stereotypes and aimed to reduce feelings of stigma and judgements every time they met with mothers [15]. Nonetheless, many of the most vulnerable mothers continue to demonstrate distrust in public health systems designed to provide them with support. Therefore, it is imperative that we understand from the perspective of first-time mothers of diverse backgrounds and situations what health and social services they need, want and believe are helpful. Healthcare providers need to understand mothers’ experiences of inclusion and exclusion related to issues of stigma and stereotypes in order to create spaces that are meaningful and accessible for new mothers and their families.

## 3. Methodology

We used the qualitative methodology feminist poststructuralism, which is “a philosophy, theory and methodology that focuses on understanding relations of power through discourse analysis” [45,46,47,48,49]. Feminist poststructuralism enabled an exploration and examination of how first-time mothers’ experiences searching for and accessing postpartum supports were socially and institutionally constructed through different subject positions such as gender, mother or ‘at risk’. Feminist poststructuralism also enabled us to examine and understand how relations of power influenced how first-time mothers searched for and navigated access to postpartum support and information in their communities and different agencies and organizations. Discourse analysis [45,46,47,49] is an approach to analysis consistent with feminist poststructuralism that was used to deconstruct the meanings of mothers’ personal experiences and how they were related to social and institutional beliefs, values and practices. This allowed us to explore and understand the relations of power that influenced how first-time mothers chose to access or not access support groups, programs and services (both informal and formal). In feminist poststructuralism, power is understood as relational in that all people have the capacity to use their power, versus a more traditional understanding of power as unidirectional and oppressive [48].

In qualitative research, the research team needs to be reflexive of who they are and how they collect and analyze the data. We had a large team comprised of senior and junior researchers who were nurses and healthcare professionals, had experience providing care for mothers postpartum and some had worked with women who had children removed by Child Protection Services. There was also an academic journalist and graduate students. Many of the team members had been conducting research together for more than 10 years focused on maternal and infant health.

### 3.1. Participant Recruitment and Data Collection

Ethics approval was obtained through the IWK Health Centre research ethics board (reference # 1020220) before beginning the research. The study was open to all Nova Scotians self-identifying as first-time mothers who were within one year of birthing or adopting a baby. Mothers had to be able to speak, understand and write English. Study posters were distributed at family resource centers, primary care offices, local libraries and a hospital, as well as shared on our research website (www.mumsns.ca) (accessed on 12 November 2021), Twitter and Facebook accounts. We used purposive sampling to recruit 37 participants from across Nova Scotia in 2016, who participated in focus groups (*n* = 19) and online questionnaires (*n* = 18). We also analyzed postings from mothers (*n* = 21) in a Nova Scotia online chat space. Therefore, the total number of participants across the study was 58. Findings from focus groups and online chats can be found in other published articles [15,16]. Since the analysis of one focus group was very different from the rest, we decided to publish these findings separately in this article. This particular focus group included 7 participants, with 5 having had experienced involvement with and/or the removal of their babies from their care by Child Protection Services. All 7 women in the focus group had a chance to speak and share their experiences. Therefore, this paper reports findings from data collected at this one family resource center. We also decided to publish findings from this focus group after publishing findings from the rest of the study in order to provide context. Eligibility requirements were met by all participants as they could speak English, were 18 years or older and had a baby 6 months or younger. Demographic information emerged from the focus group if each participant chose to share this information in the telling of their story.

Potential participants were recruited through the family resource center as staff told mothers about the upcoming focus group over the course of a few weeks prior to the date. Two of the team members, including the principal investigator and research assistant, who were gender presenting female, conducted the focus group and did not know any of the women. Participants were given time to ask questions and were told they could leave the session at any time with no repercussions. Verbal and written consent were obtained. One qualitative semi-structured interview guide was developed for the study and used across all focus groups to facilitate group discussion. Questions were open-ended to encourage participants to share what was meaningful and important to them in their own words. Sample questions included: (1) Tell us about your experiences looking for support and information after your babies were born. (2) How did that experience make you feel? The focus group lasted 60 min and was audio-recorded and transcribed verbatim with identifying information removed. See Aston et al. [15] for full details on methods. Field notes were written immediately after the focus group to capture impressions and thoughts about the session. All participant names were changed and all identifying information removed to ensure confidentiality. We chose the pseudonyms Jean, Sophie, Cheyenne, Ashley, Kara, Emma and Natalie rather than numbers as we believed that this more human element would better represent the sharing of personal stories told by participants.

### 3.2. Data Analysis

Feminist poststructuralism and discourse analysis were used to analyze the data. This is an appropriate methodology to use with a small sample of 5 as analysis seeks to first understand how personal experiences are impacted by social and institutional discourses in an in-depth way, followed by a focus on common themes between participants. Saturation is not an expectation of this methodology, nor is generalization. Rather, transferability of findings is important in qualitative research where findings make sense and may be seen in others’ everyday experiences. A guide outlining the application of feminist poststructuralism and discourse analysis, produced and published by the principal investigator, was used by the two principal investigators, research coordinator and four other co-investigators when analyzing the data [45]. They began with a careful reading of the focus group transcript to identify participants’ values, beliefs and practices about certain issues, followed by a deconstruction and reconstruction of how social and institutional discourses impacted their experiences. This included a focus on how relations of power were negotiated through participants’ subjectivity and agency [45,46,47,48,49] through language and meaning. Meetings were held to support critical discussions about emerging themes that eventually led to consensus about the findings.

## 4. Findings

Seven first-time mothers who had received support from a local family resource center attended our focus group at the center. Most of the mothers had not met before the focus group. Of these seven mothers, five had experienced contact with Child Protection Services, with four of them recently having had their babies taken into care. All the mothers told us they were first-time mothers because at the time of the focus group, they had recently given birth to a baby within the past 6 months whom they were caring for or had cared for before having had their babies taken into care by Child Protection Services. For some, it might have been their second time birthing a baby but their first time ‘being a mother’ from their perspective, and we honored their point of view with this inclusion criteria.

As the mothers shared their stories about searching for information and support, it became evident that dealing with Child Protection Services was a significant and predominant part of their experiences. While some participants shared the reasons why their babies were taken from them by Child Protection Services, as detailed in the findings below, they mainly spoke about their experiences of seeking information and support postpartum, because that was the question we asked. The mothers shared their stories openly within the focus group, revealing details that we had not anticipated, thus demonstrating the effectiveness of using qualitative feminist poststructuralism methodology and non-hierarchical interviewing skills. Three members of the research team provided food and drinks and facilitated the discussion. The findings are organized into five themes: (1) We are Mothers, (2) Being Red Flagged, (3) Lack of Trust, (4) Us Against Them and (5) Searching for Supportive Relationships.

### 4.1. We Are Mothers

The mothers who had experienced their babies being removed from their care by Child Protection Services all spoke about feeling disregarded by healthcare providers and social workers both at home and in the hospital, and that it was important to them to be recognized as mothers even though they did not have their babies with them. Sophie said she felt ignored in the hospital and she believed she was not treated by the nursing staff as well as the other mothers who had their babies with them. She had experienced a type of ‘othering’ because her baby was not with her. She said that she did not receive the ‘Loving Care Books’, which is a healthcare resource published by Public Health containing information about baby care, that all new families are to receive. She recalled that when she was crying and asking for help, she felt ignored by the nurses.

“They’re [mothers who had their babies taken away] supposed to be given everything that you would give a mother that had their baby…even if we wake up crying and hysterical in the middle of the night cause we’re upset and stuff and we ring the buzzer for medication or something I noticed they had to rush faster to the mothers with the little, tiny ones.”

(Sophie)

Sophie also said that hearing the cries of other babies ‘killed’ her and she suggested that mothers like her who have had their babies removed from their care should receive postpartum care in a room away from the sound of crying babies. In this instance, Sophie used her agency as she questioned and challenged practices within the healthcare system to be more supportive of mothers whose babies are not in their care and acknowledge them as mothers.

Natalie, who also had her child removed from her care, stated that while she was in the hospital no one checked in with her about her mental state. She shared that “I think someone coming and checking on your mental state…I would’ve loved to have someone say how are you feeling?”. Kara had a similar experience. She reflected:

“I find a lot of them [nurses], you know, are not very sympathetic, they don’t want to help you…I’m finding they need to train their staff more to be more understanding of us not just as parents and mothers, but as human beings, you know, how to interact with us with any situation and be more helpful when we’re asking questions instead of judging us or looking at us weird.”

(Kara)

Members of the focus group shared the common experience of feeling mistreated, and experiencing distrust, by healthcare providers because they had their babies taken. The meaning of mothering has been socially constructed through dominant discourses that often include ideals of women selflessly giving themselves to their babies. There has been much written about the ‘good mother’ discourse that includes stereotypes and social beliefs about how mothers should act [50,51,52,53]. The participants in this study spoke about not even being recognized or treated as mothers; thus, not fitting into normalized expectations of mothering or motherhood. Their stories highlighted how institutional and individual practices perpetuated dominant stereotypes and stigmas surrounding ‘good’ mothers and mothering practices.

### 4.2. Being Red Flagged

All of the participants spoke about feeling judged by workers in the health and social care systems, which they referred to collectively as ‘the system’. Many said they were ‘red flagged’ in pregnancy by Child Protection Services as unable to take care of a baby, either because of lifestyle choices or family histories. Cheyenne recalled: “I was into some kind of bad lifestyle choices. So, there was certain reports on me that were made but I was already red flagged”. People who are ‘red flagged’ by Child Protection Services have what is called a ‘birth alert’ attached to their health records—when they arrive at the hospital for labor and birth, the healthcare providers are required by law to contact Child Protection Services (personal communication, Paynter).

Cheyenne said she had already experienced having her first baby taken by Child Protection Services and believed that they would take her second baby from her. Canadian researchers have found that the experience of the removal of a child is associated with decreased care-seeking in subsequent pregnancies [6]. Cheyenne described how she denied a Child Protection Services worker access to her home during her pregnancy, clearly challenging a perceived threat of surveillance. Cheyenne believed Child Protection Services would inevitably take her baby from her due to past experiences with Child Protection Services and therefore she did not want to go through needless judgement. She also spoke about how she had been taken from her own mother when she was a young girl and believed that the Child Protection Services workers were judging her on the past history of her family, rather than focusing on her capabilities as a mother. Due to her experience, and what she described as workers having documented this in her file, Cheyenne did not plan or prepare to have her baby with her after the birth. When the Child Protection Services workers came to the hospital to tell her she could keep her baby, she was shocked. Cheyenne further recalled:

“They were bringing up all these things my mum did when I was younger and that I was taken for ‘cause that has nothing to do with me. I’m not my mum. Just ‘cause my mum did those certain things doesn’t mean I’m going to… He [first baby] was taken in the hospital. I never did get him back. He was adopted last summer so, when I had her [second baby] a week ago, I didn’t even have an outfit to put on her ‘cause I’m still red flagged at hospital, so I figured they would come and take her.”

(Cheyenne)

Other participants had similar experiences of intergenerational Child Protection Services involvement. Kara said Child Protection Services workers were going to take away her baby because of her mother’s overdose and Ashley also said Child Protection Services workers used her mother’s circumstances to take her baby away.

“The Child Protection Services try to use it against us, that me and my partner, that they try to use our family history saying that I was already in care…then that’s the reason why they took the child is because of my family that was going through Child Protection Services and everything else years prior to that.”

(Ashley)

Ashley also described feeling judgement for having mental health issues and a learning disability and believed Child Protection Services used this against her.

“They tried to use a learning disability against me ever since my two kids were taken from me. And they used that against me saying that I’m not capable of looking after my child just because I got a learning disability too. So, they tried to use that and the mental health against me too. And they still use it right now to this day of trying to get my child back.”

(Ashley)

Kara similarly stated “…they’ve pulled up all my past history from when I was in my mother’s care and everything and they were like this is pretty much the reason why you can’t take care of your child”, while another participant, Emma, recalled advocating against ‘the system’ with the help of her sister, stating: “mental health should have nothing to do with your kids. They tried to pull that on me too and my sister helped me fight it.”

Many participants said that personal details about their lives and their families’ lives had been recorded by Child Protection Services workers and kept in a file. They believed this information was most often used ‘against them’ rather than used in a way that was helpful to their situations. Historically, the healthcare system has been influenced by a discourse of surveillance [10], and while surveillance can be presented from the position of caring, this discourse can also be experienced as a threat to ensure compliance of patients or clients, particularly for Black persons, Indigenous peoples and People of Color. Through the discursive practice of institutional surveillance, power and control by healthcare workers is perpetuated.

### 4.3. Lack of Trust

All the participants who met with Child Protection Services spoke about a lack of trust they felt towards Child Protection Services workers and ‘the system’ in general. The mothers who experienced the removal of their babies felt they were expected to follow specific but unwritten and ever-changing rules in order to have their babies returned to them. They spoke about entrusting Child Protection Services workers for guidance and support, particularly access to mental health services and counselling, only to then experience disappointment with how they were treated through the withholding of information and supports by Child Protection Services. For these mothers, the ‘system’ created further barriers that greatly impeded their efforts to have their babies returned to them.

Sophie said Child Protection Services did not deliver on promises of services that would have included counselling and therapy. She believed Child Protection Services did not follow through on promises and this led to a lack of trust. Jean also agreed that Child Protection Services did not follow through on promises. Most participants believed that Child Protection Services workers were the only people they could access for support. To prepare to have their babies returned, participants had to put their trust in Child Protection Services workers to help them find services such as early parenting groups and counselling. When Child Protection Services workers were unable to follow through with promises, it broke the participants’ trust. Sophie reflected:

“Child services…to this day 15 months later have yet to provide me with anything they’ve claimed they were going to provide for me. Even in court they told the judge they were going to provide this list of services and I’ve seen zero of that.”

(Sophie)

Sophie also said that there were inconsistencies across various case workers where different assessments were made on situations that appeared to be similar. She concluded by stating that the Child Protection Services workers were ‘impossible’ and she ‘could not trust them’.

Kara also described losing trust in the system. Kara’s daughter was taken away after the Child Protection Services worker had originally told her they would help Kara with a plan to keep her. She recalled:

“[Child Protection Services] said that they were going to come back with a plan and then they came and took her. So, I’m still working on that which kind of scares me because I don’t even know what to do, I’ve been going here [family resource center] trying to find out what to do on my own.”

(Kara)

Cheyenne also said she felt the system was against her because Child Protection Services workers were not providing the necessary supports for her to regain custody of her son. She said Child Protection Services workers told her what she had to do but they did not link her to any of the promised services, requiring Cheyenne to seek supports on her own. The delay in accessing supports initialized by the lack of guidance or connection from Child Protection Services shortened the time she had to “prove” herself according to Child Protection Services expectations. Once Cheyenne realized the Child Protection Services workers would not fulfill their promises, this created a situation of distrust—she reflected:

“He was taken in the hospital. I never did get him back. He was adopted last summer…they take a long time to get you set up with services. By the time they do, I only had a year to prove myself to them to get my son back and it was six months before I even got setup with certain services so that gives me six months to try to prove myself which isn’t a very long time. But this time I’m just kind of doing everything on my own, just—I don’t know, everything that they [Child Protection Services] kept telling me, ‘oh we want you to take this, we want you to take that’. And I just—I’m taking everything on my own.”

(Cheyenne)

The majority of mothers described the system as restrictive and punitive. Having a 12-month window to complete required activities such as parenting and counselling sessions was seen to be a systemic barrier, especially when it was often reduced to half that time while mothers waited for support and direction from Child Protection Services workers who could not help them. Most mothers said that they did not feel they had a ‘voice’ to challenge the system as there was a sense of fear that if they did, their baby would be placed in permanent care based on what Child Protection Services workers would say against them. Based on what was shared by the participants, our interpretation was that participants had been subjectively positioned as clients in an institutionally constructed binary relation of power that entailed surveillance, documentation and judgement, where they felt they had minimal control. For these participants, surveillance had been constructed as punitive, with an intent to search only for problems with their mothering practices. It was difficult to have trust in this type of institutionally constructed relationship. Cheyenne stated:

“I just feel like they’re [Child Protection Services] going to use it against me even more for expressing the way that I feel and the way that I feel they treat people… [I’m] scared to even say anything because they use so much against you.”

(Cheyenne)

Participants had to continuously challenge social norms about what it meant to mother. They had to negotiate their subject positions repeatedly in an effort to prove to Child Protection Services their capacity to parent. Binary relations between participants and Child Protection Services workers created situations whereby participants had to negotiate power imbalances. Not only did they have to prove they were mothers, they also had to navigate through socially constructed discourses of what constitutes ‘good’ and ‘bad’ mothers. For example, mothers that were ‘red flagged’ due to a perceived inability to care for a baby and the requirement to take parenting classes and counselling before having their baby returned were situated within a ‘bad mothering’ discourse and related subject position. To keep their babies, participants had to navigate Child Protection Services’ expectations and demands to not only be seen as mothers but as ‘good mothers’.

Many mothers said Child Protection Services called into question their mothering abilities over circumstances and issues they felt were beyond their control. Mothers believed they were judged unfairly over issues they believed to be irrelevant to their ability to mother, such as past mental health issues, family members’ mental health issues or criminalization, and previous involvement with Child Protection Services as a child themselves. The mothers in this study questioned the ability of Child Protection Services workers to assess their individual situations and believed that Child Protection Services workers chose to primarily focus on external factors rather than their capacity to care for their babies. As an example of this, Kara recalled:

“They [Child Protection Services] were first involved when me and my mum got into an argument and then they were out of our lives, you know, they just wanted to make sure that everything was okay and then all of a sudden because I had one bad day with my depression and my psychiatrist at Reproductive Mental Health is extremely busy. I wasn’t on medication, I wasn’t able to see them. They judged from my past from when I was in foster care…”

(Kara)

Similarly, Emma described a situation where she was pre-judged by Child Protection Services based on her living arrangement with a parent experiencing addiction:

“Oh, my mom…she tried to OD when I was living with my grandparents upstairs but it’s like, it’s two different places and they were going to take her away from me because of that when I was living upstairs and she was living downstairs. Two different places. But when she came, she looked around and she seen what my baby had, like, she has more than enough and she was like, and she came and she was like I don’t even know why I’m here.”

(Emma)

These mothers discussed at length the situations in which they encountered adversity and judgement. They responded to each other’s stories with comments such as, “they tried to pull that on me too…” or “they tried to use that against me too,” suggesting that these experiences were universally shared. Their use of the word ‘tried’ and examples of questioning practices within Child Protection Services demonstrates the participants’ strength and agency, which informed their practices to continue searching for the necessary information and support to guide them through the postpartum period through alternative sources such as the family resource center, a non-profit organization in the community.

### 4.4. Us against Them

The participants provided many examples of adverse relationships between themselves and Child Protection Services workers. Sophie gave an example of how she felt threatened when a Child Protection Services worker screamed at her when it was only the two of them in a room together, recalling:

“I got attacked by a worker because of a very, very traumatic event in my past. They targeted me and screamed in my face…Like, screaming, not even two feet from my face. She got probably this close to my face and screamed at me. And this was a bigger woman too, probably double my size at least and I was on my own because she wanted it one-on-one.”

(Sophie)

On a second occasion with Child Protection Services workers, she knew she had to protect herself, so she audio-recorded the conversation.

“I had an audio recording going in my own home because I knew that, that way it was legal. They screamed, hollered, belittled and attacked me the second they thought I was alone when I had someone else just in another room where they did not know that other person was there. The other person came out and they were like ‘um, excuse me I was taking a nap and I got off work and I came over here to see [Sophie] and the baby. Why are you screaming at her, telling her all these lies? I’ve known her for 10 years.’ And they kind of had a bit of a—they were stunned, shocked and turned around and left as soon as they came out and said that.”

(Sophie)

This is an example of how Sophie challenged a binary relation of power between herself and a Child Protection Services worker. With the support of a friend, she was able to use her agency to negotiate the power differential between Child Protection Services and herself. To prevent further antagonism and abuse, Sophie had a lawyer intervene to ensure no further one-on-one meetings with Child Protection Services would take place.

Kara also spoke about how differently she experienced Child Protection Services’ involvement when the worker was supportive. She said some workers were understanding and others were not. Kara stated:

“The first two workers that I had, they’re really understanding things…They went and they talked to my psychiatrist, my clinical social workers, to try to understand as a person, trying to understand how my depression is, how it’s affecting me mentally and physically as well.”

(Kara)

Kara went on to say that her new Child Protection Services workers were not as supportive and felt that they might force her to take medications: “I have to get my psychiatrist stupidly enough to do a med evaluation otherwise they’re going try and force medication on me. Which I don’t think is right to do.”

Kara also said that when things went well, she felt “lucky not to be judged,” clearly indicating a position of always having to protect herself. ‘Us against them’ had been created through institutional discourses that had to be navigated by the mothers when interacting with Child Protection Services workers.

### 4.5. Searching for Supportive Relationships

All of the participants who experienced Child Protection Services’ involvement expressed experiencing some form of judgement and adversity from health and social services personnel, especially Child Protection Services workers. This came through in their stories of not being recognized as a mother, being red flagged and lacking trust in the system. They talked about the importance of having support during pregnancy and the postpartum period. After experiencing a lack of support from some healthcare providers, most said they had to search for support and guidance on their own to be able to meet Child Protection Services’ requirements to get their babies back.

All of the participants struggled to find required support during the postpartum period, whether they had custody of their babies or were working to have them returned. Cheyenne believed that the system was setting her up for failure: because Child Protection Services failed to provide support and information, she would never be able to meet Child Protection Services’ expectations and demands. She said she had to go to the family resource center on her own for guidance about how to get her child back, “I just signed up for three different programs through this place [family resource center].” She said accessing support at the family resource center was simple and felt that the family resource center offered helpful programs and provided her with the opportunity to sit and talk with staff.

“I find the center here, they really provide a lot of programs about certain things…like that they can sit down and you can set up an appointment and talk to them about certain things. And that’s really helpful, too.”

(Cheyenne)

Sophie said she knew she had to do something on her own to get her baby back, so she found the family resource center, which she further described as a form of “security blanket”:

“I found family support workers for the parents, and all kinds of parenting classes to help educate parents which all looks good in Child Protection Services’ eyes, but also looks great and very helpful for a parent. You get to socialize, and it helps you actually be free to talk to people when you have the consents and everything. So that way it was almost like a security blanket I found…It was more because there were people to talk to that I knew weren’t going to reveal everything I was saying, and people that understood. The staff at the center understood the child services the way it works, obviously having dealt with for and many clients who have dealt with it. They were able to help me navigate that sea of child services disasters very well.”

(Sophie)

Positive relationships were experienced between all the participants in our study and staff at the family resource center. They used words such as trust, confidence, support, security, non-judgmental and helpful to describe their experiences and interactions. However, many of the mothers had not been told about the family resource center by hospital staff or Child Protection Services workers, and often they had to seek out services on their own. Cheyenne said: “I got, some one-on-one counselling [inaudible] and this place is actually, really awesome. It’s a really big help I think to new mothers and stuff.”

Similarly, Kara said:

“The only one that actually had resources is here…And that’s what I found out with this center here, they sit there one-on-one and they sit there and talk to you, and even though they have a program here…and they find the resources that you need outside of the community center. They sit there and they help you with all the staff here so, yeah.”

(Kara)

Natalie appreciated the help she received from the family resource center and enjoyed being able to socialize with others so that they could tell her what she was experiencing was normal: “Just hearing other stories and not feeling so alone in what you’re going through and I think some of the other classes that I’ve taken here I see some of the mums and they have everything so put together.”

All the participants in our study demonstrated strength as they navigated the healthcare and child welfare systems. They had to negotiate many relations of power as they were often positioned as either non-mothers or bad mothers that had been constructed socially and institutionally. They had to search for supportive people and organizations that would help them navigate the system, as Sophie clearly stated: “They [family resource center staff] were able to help me navigate that sea of child services disasters very well.”

## 5. Discussion

The aim of the study was to understand how new mothers in Nova Scotia prioritized their postpartum needs and where they went to obtain information and support. As mothers involved with Child Protection Services, all the participants described experiencing some form of stigma from workers in ‘the system’ and this impacted their efforts to search for postpartum support and information to help them retain or regain custody of their infant. The ‘system’ is the connection between the healthcare institution where they gave birth and had their children removed from their care by Child Protection Services and continued surveillance and control by Child Protection Services while negotiating the return of their children. For these participants, the prospect of having their children returned to them was motivation to seek out supports for their identified needs (e.g., mental health). However, Child Protection Services as an institution clearly caused harm to these new mothers, initiated by the trauma experienced when their child was removed based on, as the mothers described, biased or historically perceived risks regarding one’s ability to parent, direct verbal abuse by workers from this institution and in the creation of a cycle of unreasonable and unclear expectations of parenting, coupled with a lack of information and support from these same institutions.

Child Protection Services has been institutionally constructed as a risk-averse service [54,55]. Assessments and decisions by Child Protection Services workers to remove infants and children are guided by institutional guidelines. Focus is primarily on the safety of the child that may include either providing supports and services to parents to keep their child in care or removal of the child from their parents [56]. Findings from our study included mothers’ personal narratives of children being taken from them in the early postpartum period that appeared to be based on assessments of potential risk of abuse or neglect. All participants told us that in order to keep their child or have their child returned to them, they were required to follow an unclear plan with ever-changing requirements, none of which were achievable. Expectations to find supports on their own, yet feeling unsupported by healthcare workers, weighed heavily on them emotionally and mentally and affected their experiences of searching for information and support. Competing social discourses based on beliefs and practices of mothering made their experiences of navigating the healthcare system even more challenging as they were ‘red-flagged’, felt that they were seen as ‘bad’ mothers and were ultimately labeled as ‘at risk’.

Canadian researchers conducted a study with 1000 participants and found that the removal of children by Child Protection Services was associated with a 55% increased risk of non-fatal overdose [57], with the risk nearly doubling among Indigenous women. They concluded that removal of a child at birth may cause a mother to relapse and return to substance use. In a systematic review, Marsh and Leamon [58] concluded that similar to the death of a child, the removal of a child from custody has extreme psychological effects including grief and guilt. Healthcare workers need to understand the impact of the trauma that is inflicted on mothers whose infants are removed at birth so that they can effectively support them in their journeys to ‘be mothers’ and find caring supportive people and environments to help facilitate the return of their children. Mothers in this study experienced a lack of supportive care. Mothers being separated from their babies and not supported with opportunities to demonstrate how they were ready to have their babies returned may be seen as a failure by Child Protection Services [59]. We recommend that before reporting women to Child Protection Services, healthcare workers ensure that their assessments are evidence-based with a focus on demonstrated harm.

Being labeled as ‘at risk’, ‘vulnerable’ or ‘marginalized’ is based on health assessments about mothers who might be experiencing lower socioeconomic status, domestic violence, mental health issues, being a single parent or dealing with Child Protection Services in the past, which is meant to be helpful. However, this labeling also contributes to the ongoing negative stigma and stereotypes that continue to be socially constructed for mothers and unjustly contributes to increased referrals to Child Protection Services. For example, the number of Black and Indigenous children in foster care is disproportionately high in Canada. Although only 7% of children in Canada are Indigenous, Indigenous children make up 52% of children in foster care [60]. As the Restorative Inquiry into the Home for Colored Children demonstrated, Nova Scotia has a long history of over-surveillance of African Nova Scotian families, and Black children are over-represented in the child welfare system in this province [60]. Racism and colonialism are foundations of the child protection regimes in Canada.

Mothers in this study told us how they were ‘red flagged’ while in the hospital before giving birth. This practice is also known as a birth alert and has been banned in most of Canada; however, is still permitted in Nova Scotia [61]. Birth alerts have been condemned as discriminatory and targeting Indigenous and Black mothers [7,8]. Birth alerts are when hospitals and social services document mothers as ‘high risk’ and ‘red flag’ them based on a parent’s history, including poverty, domestic violence, substance use or a history with Child Protection Services. This practice is usually carried out without the parent’s consent. Knowing that this practice is controversial and is being challenged in Nova Scotia, where the participants in our study reside, requires us as researchers and healthcare professionals to continue to challenge the meaning of ‘at-risk’ parents that perpetuate harmful stereotypes. We need to find better ways to support parents. The Canadian government [23,24,27] states that postpartum care should be available to all mothers and families, and yet navigation and access to postpartum support that will enable mothers to keep their children is unclear and often impeded by Child Protection Services’ intervention.

The participants in our study clearly articulated the harms that they experienced while in ‘the system’ and used this to question and challenge Child Protection Services’ practices. Support was imperative—they found it through their own agency, usually through the assistance of non-governmental, non-profit, underfunded community organizations.

## 6. Limitations

While in-depth interviews in a focus group with a select group of participants is considered a strength of qualitative research, it may also be considered a limitation as findings are not generalizable. Therefore, we would suggest that more studies be conducted with parents who are being monitored by and/or who have had their infants taken into care by child protection services. Except for eligibility criteria of age, English speaking and being 18 years or older, we chose not to collect specific demographic data trusting that participants would share relevant demographic information if it related to their stories such as race, income or gender status. We were able to analyze participant experiences without this information; however, recognize that some may view this to be a limitation and therefore suggest that future studies include demographic data as part of larger studies that may want to generalize findings. This study is based on a single focus group, located at the family resource center, where the participants stated they finally found needed support. Although no family resource center staff were present in the focus group, participants may have felt they needed to describe the family resource center services positively.

## 7. Conclusions

Mothers in this study did not differentiate between healthcare providers in the health system, such as nurses, and child protection workers. Traumatic experiences with Child Protection Services and the surveillance they are under by healthcare providers resulted in mistrust of both. Despite needing to meet Child Protection Services’ expectations to be reunited with their children, by addressing issues such as mental health or parenting skills, resources were not provided by Child Protection Services to these mothers and they were therefore left to their own devices to seek support. Government departments such as health and Child Protection Services have downloaded postpartum and parenting support onto non-profit community organizations. Participation in programs at these sites is somewhat performative, done simply to try to assuage Child Protection Services. The dominant postpartum need for these mothers is reunification. The mental and emotional anguish of child removal is not treated with care and compassion by healthcare workers. Due to the over-representation of Black and Indigenous children in foster care in Nova Scotia, urgent attention is needed to how these systems perpetuate colonialism, racism and genocide.
